# Artificial Photothermal Nociceptor Using Mott Oscillators

**DOI:** 10.1002/advs.202409353

**Published:** 2024-12-18

**Authors:** Pyeongkang Hur, Daseob Yoon, Minwook Yoon, Yunkyu Park, Junwoo Son

**Affiliations:** ^1^ Department of Materials Science and Engineering Pohang University of Science and Technology (POSTECH) Pohang 37683 Republic of Korea; ^2^ Department of Electrical Engineering Pukyong National University Busan 48513 Republic of Korea; ^3^ Department of Materials Science and Engineering Seoul National University Seoul 08826 Republic of Korea; ^4^ Research Institute of Advanced Materials Seoul National University Seoul 08826 Republic of Korea; ^5^ Institute of Applied Physics Seoul National University Seoul 08826 Republic of Korea; ^6^ Materials Science and Technology Division Oak Ridge National Laboratory Oak Ridge TN 37830 USA

**Keywords:** artificial nociceptor, metal‐insulator transition, neuron, oscillator, oxide

## Abstract

Bioinspired sensory systems based on spike neural networks have received considerable attention in resolving high energy consumption and limited bandwidth in current sensory systems. To efficiently produce spike signals upon exposure to external stimuli, compact neuron devices are required for signal detection and their encoding into spikes in a single device. Herein, it is demonstrated that Mott oscillative spike neurons can integrate sensing and ceaseless spike generation in a compact form, which emulates the process of evoking photothermal sensing in the features of biological photothermal nociceptors. Interestingly, frequency‐tunable and repetitive spikes are generated above the threshold value (*P_th_* = 84 mW cm^−2^) as a characteristic of “threshold” in leaky‐integrate‐and‐fire (LIF) neurons; the neuron devices successfully mimic a crucial feature of biological thermal nociceptors, including modulation of frequency coding and startup latency depending on the intensity of photothermal stimuli. Furthermore, Mott spike neurons are self‐adapted after sensitization upon exposure to high‐intensity electromagnetic radiation, which can replicate allodynia and hyperalgesia in a biological sensory system. Thus, this study presents a unique approach to capturing and encoding environmental source data into spikes, enabling efficient sensing of environmental sources for the application of adaptive sensory systems.

## Graphical abstract

This study proposes a bioinspired approach to integrate photothermal sensing with continuous spike generation in compact Mott oscillative spike neurons. The devices replicate essential functions of biological thermal nociceptors, including frequency modulation and adjustments in startup latency depending on the intensity of photothermal stimuli, effectively mimicking allodynia and hyperalgesia phenomena in sensory systems.

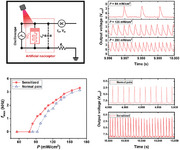

## 1 Introduction

The exponential demand for real‐time sensing and processing of environmental data calls for efficient, adaptive, and compact sensory systems in the Internet of Things (IoT) era.^[^ ^1^, ^2^, ^3^, ^4^, ^5^, ^6^ ^]^ However, conventional sensory systems are operated by the sequential processing of physically separated sensors, memory, and processing units;^[^ ^3^ ^]^ these conventional sensory devices are plagued by high energy consumption, significant latency, and limited bandwidth for real‐time imaging and high‐resolution data processing.^[^ ^7^, ^8^ ^]^ The difficulties in current sensory systems have drawn interest to bioinspired sensory neurons that emulate the human sensory system.^[^ ^1^, ^3^ ^]^ The human sensory neurons capture and integrate environmental data from their receptors, encoding spatiotemporal information into spikes (i.e., all‐or‐nothing electrical impulses) whenever relevant changes are detected. This information‐processing method efficiently addresses the limitations faced by conventional sensory systems and has inspired researchers to develop new sensory processing systems. Notably, spike neural networks (SNNs), which mimic the information processing mechanisms of the human sensory system, reduce energy consumption while enabling efficient sensing of multiple types of information in parallel processing involving large datasets.^[^ ^9^, ^10^, ^11^, ^12^ ^]^

Recently, memristive receptors and peripheral spike encoders compatible with SNNs have been combined to replicate data processing in afferent nerves in biological sensory systems, which inherently perform parallel data processing.^[^ ^13^, ^14^, ^15^, ^16^, ^17^, ^18^ ^]^ However, the peripheral spike encoders, such as op‐amps and Schmitt triggers, need to be more compact and efficient due to their large area and high energy consumption, respectively.^[^ ^2^, ^16^, ^17^ ^]^ The use of relaxation oscillators has been suggested for spike generation due to a further simplified design and lower power consumption of the devices, but the physical separation between devices continues to be problematic in terms of energy consumption, latency, and bandwidth; the artificial afferent nerves that integrate sensing and spike encoding functions in a single device need to be developed to address these limitations.^[^ ^19^ ^]^

In addition to simple spike generation as an afferent nerve, the concept of nociceptors has been suggested to mimic afferent nerves in biological sensory systems comprehensively.^[^ ^13^, ^14^, ^15^, ^20^, ^21^ ^]^ Unlike other sensory systems, nociceptors discern harmful stimuli and trigger self‐protective responses via spike transmission to the central nervous system, which is crucial for survival: Nociceptive neurons evaluate stimuli from receptors, selectively encoding intense and harmful signals into spikes, which are transmitted to the brain for appropriate responses.^[^ ^22^, ^23^ ^]^ Artificial nociceptors have been emulated using memristive devices. Still, these devices either lack the capability to sense external stimuli other than electrical signals or produce analog output signals instead of spikes, making them impractical for application in SNN‐based sensory systems.^[^ ^4^, ^24^, ^25^ ^]^ Moreover, it would be beneficial to develop a compact nociceptor device that can detect external stimuli and encode signals into spikes within a single device.

Herein, we demonstrate a bioinspired photothermal nociceptor using Mott oscillative spike neurons that integrate sensing and spike encoding functions into a single device. A two‐terminal VO_2_ threshold switch absorbs the light, and then the switches connected in series with a resistor generate ceaseless spikes as relaxation oscillations based on the charging and discharging process. Interestingly, frequency‐tunable and repetitive spikes are generated when exposed to infrared radiation with different power intensity, exceeding the threshold value (*P_th_* = 84 mW cm^−2^) as a characteristic of “threshold” in leaky‐integrate‐and‐fire (LIF) neurons: the generation of spikes in our photo‐thermal‐triggered VO_2_ oscillators above *P_th_* successfully emulated a crucial feature of biological thermal nociceptors, including modulation of frequency coding and startup latency with increasing the intensity of thermal stimuli. As an environmentally adaptive response, the artificial Mott nociceptor is sensitized after exposure to high‐intensity electromagnetic radiation, which can replicate allodynia and hyperalgesia in a biological nociceptor.

## 2 Results and Discussion

**Figure 1:**
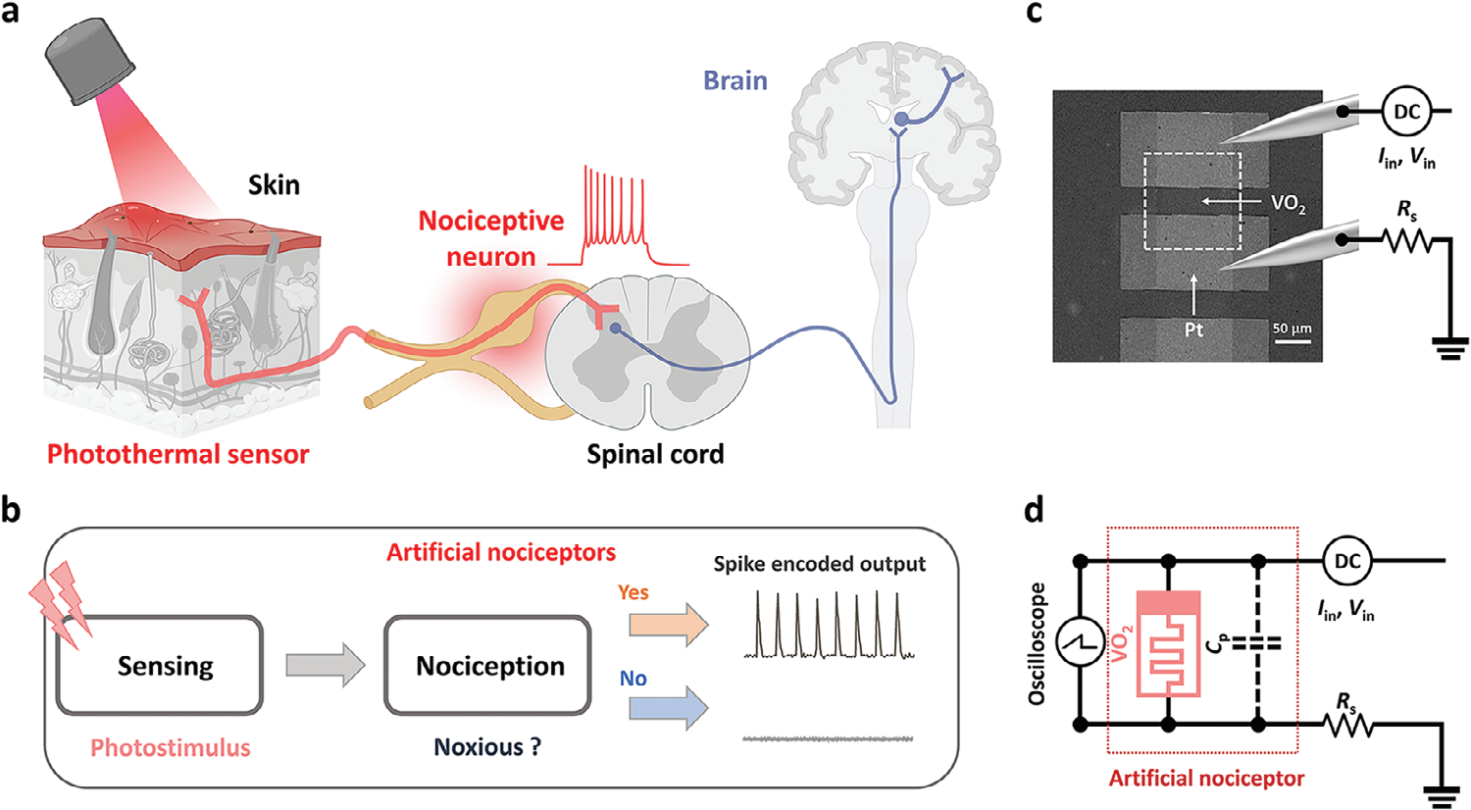
Demonstration of bio‐inspired artificial nerves using Mott oscillators. a) Illustration of a biological nociceptor: When a stimulus is received from a nerve ending, the nociceptive neuron compares the signal's amplitude with a threshold value, deciding whether to generate an action potential and send it to the cortex via the spinal cord. b) the artificial nociceptor for detecting electromagnetic radiation: Upon illumination, the artificial nociceptor detects light and generates a spike only when the light intensity is high enough to trigger the oscillator. c) Plane‐view scanning electron microscopy (SEM) image of VO_2_ threshold switch connected in series with a resistor. d) Oscillative spike neurons based on Pearson‐Anson circuits consisting of a VO_2_ threshold switch, a parasitic capacitor, and a series resistor.

The principles of the human sensory systems for detecting electromagnetic radiation (light) correspond to artificial afferent nerves manifested by VO_2_ Mott oscillators (**Figure** **1**a,b). Our bio‐inspired artificial nerves (Figure 1b) mimic their biological counterparts by directly detecting environmental stimuli and transferring this data to the cortex in the form of electrical spikes (Figure 1a). Our simple device is composed of a two‐terminal VO_2_ threshold switch connected in series with a resistor (Figure 1c,d); this device, which contains VO_2_ thin films, can function as both light‐absorbing sensors and spike generators, based on a relaxation oscillator circuit;^[^ ^14^, ^20^, ^26^ ^]^ spike‐encoded electrical output needs to be modulated by photostimuli input through our artificial nerve devices.

After successfully growing VO_2_ epitaxial films on (001) TiO_2_ substrates,^[^ ^27^, ^28^ ^]^ as confirmed in Figure S1 (Supporting Information), two‐terminal devices were fabricated with a lateral Pt electrode separation of 35 µm on the VO_2_ epitaxial films, as shown in the plane‐view scanning electron microscope image (see Figure 1c). The VO_2_ epitaxial films exhibit a temperature‐dependent metal‐insulator transition at 310 K, enabling the demonstration of threshold switching at 298 K (Figure S2, Supporting Information). The two‐terminal devices show the volatile threshold switching, attributed to voltage‐triggered insulator‐to‐metal transition during voltage sweep, with minimal cycle‐to‐cycle variation (Figure S3, Supporting Information).^[^ ^29^, ^30^, ^31^, ^32^, ^33^ ^]^ Device‐to‐device variation data are presented in Figure S4 (Supporting Information). By connecting a series resistor (*R_s_*), along with a parasitic capacitor (*C_p_*), the threshold switching characteristics can be utilized for oscillative spike neurons based on Pearson–Anson circuits (Figure 1d).^[^ ^12^, ^34^ ^]^ To ensure device stability, we employed this configuration with a series resistor, despite the memristor's ability to oscillate independently under current sourcing. Note that under current sourcing conditions, the frequency and operation range remain nearly invariant, regardless of the presence or value of the series resistor (Figure S5, Supporting Information). This configuration yielded consistent spike output over more than 10⁷ cycles, underscoring the device's high durability and its promising potential as an artificial nociceptor (Figure S6, Supporting Information).

**Figure 2:**
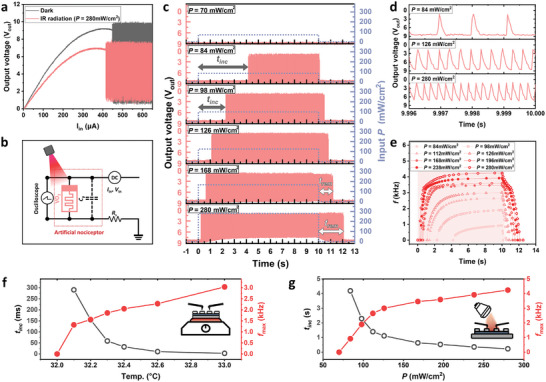
Artificial photothermal nociceptors using Mott oscillators. a) Current‐triggered threshold switching in VO_2_ in dark conditions (black line) and under exposure to infrared light radiation (red line, *P* = 280 mW cm^−2^). The exposure to infrared light radiation resulted in a decrease in the threshold current (*I_th_* = 447 µA→413 µA) and the reduction of corresponding threshold *V_out, th_* (=9.04 V→6.81 V) across VO_2_ thin films. b) Description of the artificial nociceptor circuit for light‐triggered spike oscillation of *V_out_*. c) Frequency‐tunable and repetitive *V_out_* spike generation upon infrared radiation exposure with different *P*. d) Enlarged view of spike‐encoded output at the maximum frequency (*f_max_*) from c. e) Encoded frequency as a function of time after the initiation of spike generation. The frequency gradually increased and saturated at the maximum frequency (*f_max_*). The parameters of the spikes generated (*t_inc_*, *f_max_*) in our artificial nerve systems with the increase of temperature f) and infrared radiation g). The similarity of parameters strongly supports photothermal absorption on VO_2_ films as the origin of photo‐triggered spike generation.

**Figure** **2**a illustrates current‐triggered threshold switching in VO_2_ connected with a series resistor (black line). The current sweep (0 ≤ *I* ≤ 650 µA) leads to an abrupt decrease in voltage across the VO_2_ thin films (*V_out_*) at the threshold current (*I_th_*), followed by the oscillation of *V_out_*, as observed by an oscilloscope; when the load line of the resistor in the circuit enters the oscillative region, the continuous oscillation can be detected in Pearson–Anson circuits by converting back and forth between the insulating and metallic phase of the VO_2_ films (Figure 2b);^[^ ^12^, ^26^, ^34^ ^]^ the resistance of the series resistor (*R_S_*), along with supply current (*I_IN_*) or voltage (*V_IN_*) provided through the SMU (Source measure unit), determine whether ceaseless spikes are observed as relaxation oscillation based on the charging and discharging process.^[^ ^34^, ^35^ ^]^

Interestingly, the exposure to infrared light radiation (*λ* = 750 nm ∼ 1050 nm, power intensity (*P*) = 280 mW cm^−2^), which VO_2_ channel effectively absorbs (see Figure S7, Supporting Information), resulted in the decrease in the threshold current (*I_th_* = 447 µA → 413 µA) and the reduction of corresponding threshold (*V_out, th_* (=9.04 V→6.81 V) across VO_2_ thin films, which is related to the decrease in electrical resistance before the threshold switching (red line in Figure 2a); infrared radiation facilitates the insulator‐to‐metal transition in VO_2_ thin films by adjusting the threshold value for switching (*I_th_*, *V_out, th_*) (Figure 2b). For instance, when a constant *V_IN_* (=8.6 V) is applied in the circuit, no *V_out_* spike is observed on the oscilloscope because *V_IN_* (<9.04 V) is insufficient to initiate the formation of the metallic phase. Since infrared radiation increases the conductance of the insulating phase, it is likely to assist in forming the metallic phase in the insulating VO_2_ matrix by lowering *V_out,th_* below the *V_in_*. Indeed, frequency‐tunable and repetitive *V_out_* spikes were generated when exposed to infrared radiation with different *P* (Figure 2c,d), exceeding the threshold value (*P_th_* = 84 mW cm^−2^). In this context, our device exhibits an all‐or‐nothing response, producing spikes only when the input signal reaches the threshold, which enhances noise resistance and conserves energy by remaining idle in the absence of significant input. Interestingly, a certain incubation time (*t_inc_*) is required to initiate the spike generation (i.e., startup latency) in all radiation intensities exceeding *P_th_*, which is the characteristic of “threshold” in leaky‐integrate‐and‐fire (LIF) neurons.^[^ ^14^, ^26^ ^]^ The *t_inc_* decreases with increasing *P*; higher *P* leads to faster activation of spike generation related to the perception of infrared radiation.

After the initiation of spike generation, the frequency gradually increased and saturated at the maximum frequency (*f_max_*): The *f_max_* also increased with *P* (i.e., *f_max_* = 919 Hz at *P* = 84 mW cm^−2^ → *f_max_* = 4.24 kHz at *P* = 280 mW cm^−2^ (Figure 2c–e); this characteristic of the artificial nerve system replicates the intensified perception of pain with increased radiation stimuli.^[^ ^36^, ^37^ ^]^ Additionally, under 280 mW cm^−^^2^ light exposure, the output spike rate stabilizes at 4.24 kHz from 7.14 s onward, replicating the no‐adaptation response observed in biological systems. As soon as the radiation was turned off, the spike gradually ceased in preparation for the next photostimulus, which indicates that spike‐encoded output transfer is reversibly controlled by infrared radiation input. However, remnant spike generation continued for a short period even after removing the radiation input; the duration of the remnant spike (*t_rem_*) increases with *P* (e.g., *t_rem_* = 130 ms for *P* = 84 mW cm^−2^ → *t_rem_* 2.12 s for *P* = 280 mW cm^−2^), indicating an enhanced lifetime of metallic domains at higher *P* (Figure 2c–e; see detailed view in Figure S8, Supporting Information).

The parameters of the spikes generated (*t_inc_*, *f_max_*, and *t_rem_*) in our artificial nerve systems are highly influenced by the history of infrared radiation intensity (different *P* for 10 s), which emulates the process for evoking photothermal sensing in the features of biological nociceptors. Biological nociceptors utilize different fibers to effectively transmit defensive responses to photothermal stimuli. These fibers produce outputs with varying conduction velocities and maximum frequencies, delivering the strength of photothermal stimuli based on the distinct spike‐encoded signals.^[^ ^38^, ^39^ ^]^ Similarly, our devices exhibit faster signal transmission (i.e., decreased *t_inc_*) and more activated response signal transmission (i.e., increased *f_max_*) with a higher level of infrared radiation (*P*) (Figure 2e), allowing the efficient communication of spike information regarding the level of photostimuli to the spiking neural processing unit.

The physical origin of reversible spike generation is attributed to the radiation‐stimulated decrease in electrical resistance in infrared‐absorbing VO_2_ thin films. Because infrared radiation causes temperature increases in VO_2_ thin films, this material with abrupt negative resistance‐temperature coefficient leads to a substantial decrease in electrical resistance in the VO_2_ switching layer; by facile formation of metallic phases in an insulating matrix, the threshold value for switching (*I_th_*, *V_out, th_*) decreases under infrared radiation, which enables the spontaneous spike generation. *f_max_* in the Pearson‐Anson circuits strongly depends on the *C_P_*, *R_S_* and $R_{VO_{2}}\;\left(P\right)$ in the following relationship.^[^ ^40^, ^41^ ^]^

(1) $$f_{max}\propto \frac{R_{VO_{2}}\left(P\right)+R_{s}}{C_{p}R_{VO_{2}}\left(P\right)R_{s}}$$

where $R_{VO_{2}}\;\left(P\right)$ is the *P*‐variable resistance of VO_2_ films. In fact, the decrease of $R_{VO_{2}}\;\left(P\right)$ upon radiation exposure is supported by the slope increase of the current–voltage characteristics below threshold switching (Figure 2a).

Similar to reversible control of oscillation by photon radiation, *f_max_* (or *t_inc_*) gradually increases (or decreases) with the device temperature, strongly supporting photothermal absorption on VO_2_ films as the origin of photo‐triggered spike generation: The VO_2_ device triggers spike generation at temperatures of 32.1 °C or higher, and its *f_max_* increases with the intensity of the thermal stimulus (Figure 2f). Likewise, higher *P* shortens the time required for a spike to initiate by abruptly increasing temperature (Figure 2g). In contrast, residual heat needs to be released from VO_2_ layers even after radiation input ceases, which causes persistent spike generation due to gradual thermal release.

In biological systems, thermal sensors located at the nerve endings of biological nociceptors are utilized to perceive infrared radiation;^[^ ^36^, ^42^ ^]^ these sensors naturally function as thermoreceptors, transmitting thermal pain information through two different types of nerve fibers; the generation of spikes in our photo‐thermal‐triggered VO_2_ oscillators above *P_th_* successfully emulated a crucial feature of biological thermal nociceptors, including modulation of frequency coding and startup latency with increasing intensity of thermal stimuli.

**Figure 3:**
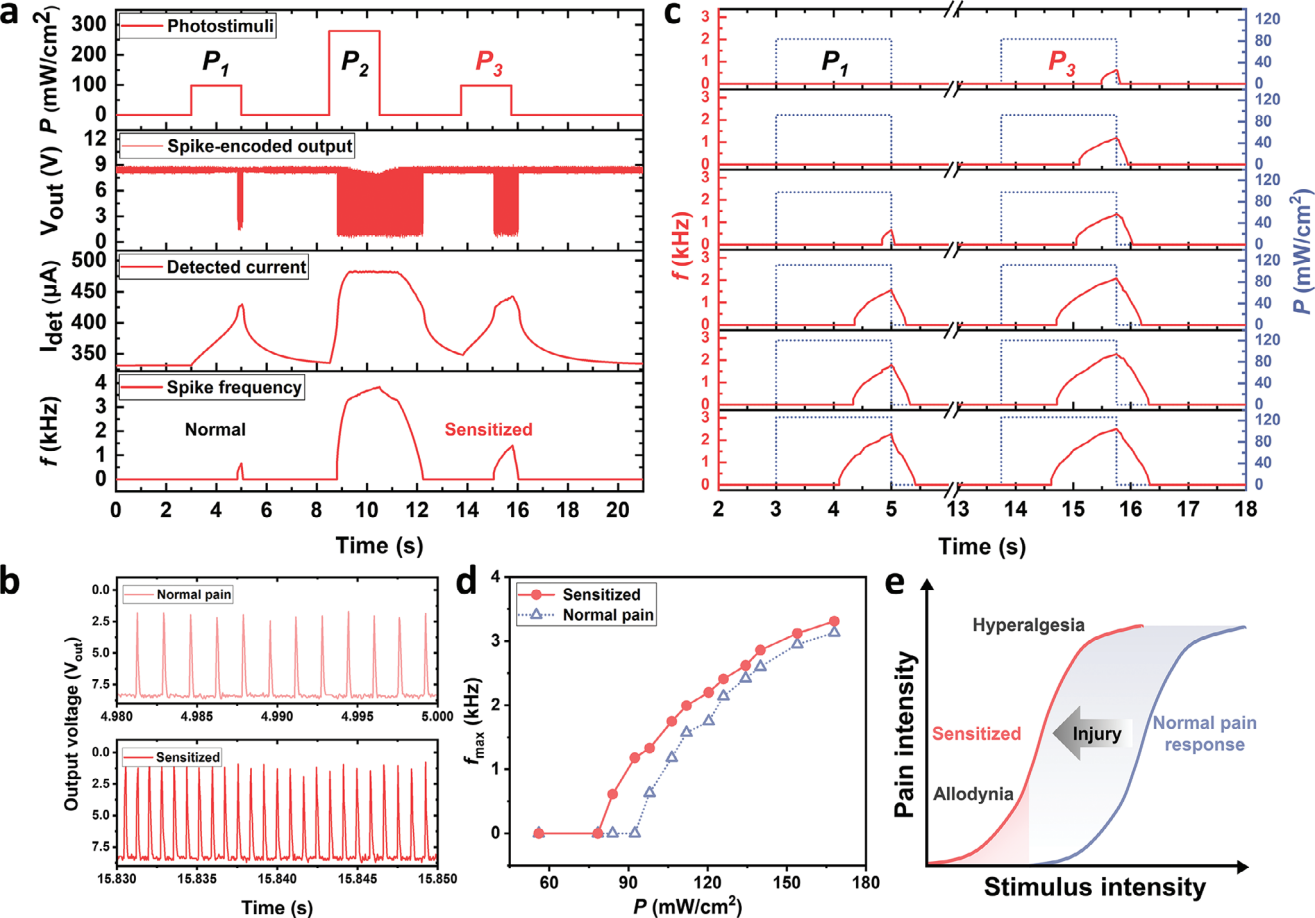
History‐dependent sensitization of our artificial nociceptor under intense photo‐stimulation. a) The spike frequency response of artificial nociceptors under three consecutive infrared pulses (*P_1_*, *P_2_*, and *P*_3_, with *P*_2_ > *P*_1_ = *P*_3_). High intensity of infrared radiation (i.e., *P_2_* = 280 mW cm^−2^) *sensitized* the artificial VO_2_ nociceptor; this sensitized nociceptor, after exposure to high radiation, strongly changes *t_inc_* and *f_max_*. b) Enlarged view of spike‐encoded output in normal and sensitized states before and after the exposure of *P_2_*, respectively. c) The spike frequency response of the VO_2_ artificial nociceptor in a sensitized state with different *P*_1_ and *P*_3_. d) More exaggerated responses in a sensitized state in the VO_2_ artificial nociceptor, which mimics allodynia and hyperalgesia in a biological nociceptor. e) Sensitization characteristics of our nociceptor (i.e., allodynia and hyperalgesia), especially these key features, align with those of the biological nociceptor.

The firing rate of the biological nociceptive thermoreceptor neuron above the threshold increases under intense stimulation at the nociceptor nerve and returns to its original state when the stimulation ceases. However, intense thermal radiation sensitizes the nociceptive neuron after the relaxation of spike firing, lowering the threshold value for spike firing.^[^ ^37^, ^43^ ^]^ This relaxation and sensitization ultimately determine the history‐dependent firing rate of action potentials in the nociceptive neuron. To replicate these features, a constant voltage with compliance current (i.e., *V_IN_* = 8.6 V, *I_CC_* = 440 µA) was applied to our artificial nociceptor, while three consecutive infrared pulses (*P_1_* = 84 mW cm^−2^ → *P_2_* = 280 mW cm^−2^ → *P_3_* = 84 mW cm^−2^) were illuminated for a duration of 2 s each, with a 3.5 s interval between *P_1_* and *P_2_*, and a 3.25 s interval between *P_2_* and *P_3_* (**Figure** **3**a).

As depicted in Figure 3a, the spike frequency increased with the intensity of the infrared radiation (*f_max_* = 655 Hz for *P_1_* = 84 mW cm^−2^→*f_max_* = 3.83 kHz for *P_2_* = 280 mW cm^−2^), indicating more sensitivity to higher stimuli. Interestingly, high intensity of infrared radiation (i.e., *P_2_* = 280 mW cm^−2^) *sensitized* the artificial VO_2_ nociceptor; this sensitized nociceptor after exposure of high radiation strongly changed *t_inc_* and *f_max_* even under identical radiation intensity (i.e, *f_max_* = 1.40 kHz, *t_inc_* = 1.30 s for *P_3_* = 84 mW cm^−2^), compared to pristine nociceptor (*f_max_* = 655 Hz, *t_inc_* = 1.84 s for *P_1_* = 84 mW cm^−2^). The origin of this characteristic can be understood through electrical analysis. *I*_det_, measured by the SMU at a low sampling rate, inversely reflects changes in the high resistance state (HRS) resistance. After *P*_2_ irradiation, *I*_det_ did not return to its original state, suggesting that the channel resistance did not fully relax due to residual heat. As a result, the oscillation conditions were more easily achieved during the *P*_3_ irradiation. The preceding intense stimuli exaggerate the pain information evoked by the third pulse, indicating that an innocuous stimulus can be perceived as painful under sensitized conditions (Figure 3b).

More exaggerated responses in a sensitized state could be observed in the VO_2_ artificial nociceptor, which could mimic allodynia and hyperalgesia in a biological nociceptor (Figure 3d). To clarify these essential features in the nociceptor, we repeated the experiment by adjusting the intensity of the first (*P_1_*) and third infrared photostimuli (*P_3_*), aiming to discern the sensitization effect on pain signals (i.e., oscillation frequency) (Figure 3c). The different pain signal under the same intensity of photostimuli (*P_1_*, *P_3_*) was detected before and after the high intensity of infrared radiation (*P_2_* = 280 mW cm^−2^). In particular, before the *P_2_* radiation, the nociceptor perceived *P_1_* < 92.4 mW cm^−2^ to be innocuous, which did not generate spikes. However, the sensitized nociceptor could generate spikes with *P_1_* > 78.4 mW cm^−2^ by lowering threshold intensity; the behavior described is comparable to allodynia in biological neurons. Additionally, the trains of spikes generated by the sensitized nociceptor exhibit a higher *f_max_* and longer *t_rem_* than those generated by the unaffected nociceptor under *P_1_* = *P_3_* > 92.4 mW cm^−2^, which is referred to as hyperalgesia; the sensitization characteristics of our nociceptor, especially these key features, align with those of the biological nociceptor, as depicted in Figure 3e.^[^ ^14^, ^15^ ^]^

To further demonstrate the artificial nociceptor under broadband photostimuli, we conducted similar experiments under UV (*λ* = 250 nm ∼ 385 nm) and visible (*λ* = 385 nm ∼ 740 nm) light radiation, as previously performed with infrared radiation. Exposure to UV (*P* = 80 mW cm^−2^) and visible light (*P* = 280 mW cm^−2^) reduced the threshold current to 435 and 414 µA, respectively, and decreased the threshold V_out, th_ to 8.16 and 6.32 V (Figure S9, Supporting Information). Similar to infrared, these wavelengths facilitate the formation of the metallic phase within the insulating VO_2_ matrix, thereby adjusting the threshold values for switching.

**Figure 4:**
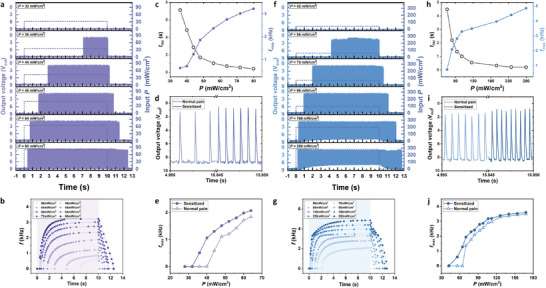
Demonstration of broadband light‐responsive artificial nociceptor. Frequency‐tunable and repetitive V_out_ spike generation upon UV a) and visible light f) radiation exposure with different *P*. Encoded frequency as a function of time after the initiation of spike generation: UV b), visible light g). The parameters of the spikes generated (*t_inc_*, *f_max_*) in our artificial nerve systems with the increase of light intensity: UV c), visible light h). Spike‐encoded output in normal and sensitized states before and after the exposure of *P_2_*: UV d), visible light i). More exaggerated responses in a sensitized state in the VO_2_ artificial nociceptor, which mimic allodynia and hyperalgesia in a biological nociceptor: UV e), visible light j).

In addition, under constant V_in_ (=8.6 V), frequency‐tunable and repetitive V_out_ spikes were generated when exposed to broadband photostimuli with varying *P*, exceeding the threshold value (*P*_th, UV_ = 36 mW cm^−2^, *P*_th, VIS_ = 56 mW cm^−2^) as shown in **Figure** **4**a,f. Consistent with the results in Figure 2e, as *P* increased, *t*_inc_ decreased, and the frequency gradually increased, eventually saturating at the corresponding *f_max_*, confirming modulation of frequency coding and startup latency depending on stimuli. (UV: Figure 4b,c; VIS: Figure 4g,h). Notably, while these trends were consistently observed across all three spectral ranges, the magnitude of changes in each parameter varied depending on the wavelength (Figure S10, Supporting Information). This feature enables selective discrimination of harmful photostimuli across various wavelengths, ensuring an appropriate response by reacting to noxious stimuli while disregarding innoxious ones.

To confirm nociceptor sensitization across all three spectral ranges, we repeated the experiments shown in Figure 3a. Following high‐intensity broadband light irradiation (*P*_2uv_ = 80 mW cm^−2^, *P*_2vis_ = 280 mW cm^−2^), the artificial nociceptor exhibited more exaggerated responses, replicating features of allodynia and hyperalgesia (Figure 4e,j). For both types of photostimuli, the nociceptor did not produce spikes at light intensities perceived as innocuous by the nociceptor in its pristine state. However, in the sensitized state, spikes were generated even at the same light intensities. Under the same UV photostimuli (*P*_1uv_, *P*_3uv_ = 44 mW cm^−2^), which exceeds the innocuous intensity, the sensitized nociceptor showed a higher *f_max_* of 1.34 kHz, compared to 582 Hz for the pristine nociceptor (Figure 4d). Similarly, after sensitization, the *f_max_* increased to 1.93 kHz under the same visible photostimuli (*P*_1uv_, *P*_3uv_ = 70 mW cm^−^^2^), compared to 1.36 kHz for the pristine nociceptor (Figure 4i).

## 3 Discussion

The above results reveal that Mott oscillative spike neurons could integrate light sensing and ceaseless spike generation in a compact form, which emulates the process of evoking photothermal sensing in the features of biological nociceptors. In particular, spikes were only generated when the light intensity exceeded a threshold value (*P_th_* = 84 mW cm^−2^) as a characteristic of “threshold” in leaky‐integrate‐and‐fire (LIF) neurons. Moreover, the generated frequency of spikes increased with the intensity of infrared radiation, which successfully mimicked a crucial feature of biological thermal nociceptors, including modulation of frequency coding and startup latency depending on the intensity of thermal stimuli. Finally, the Mott spike neurons are self‐adapted after sensitization upon exposure to high‐intensity infrared radiation, which could replicate allodynia and hyperalgesia in a biological sensory system. Thus, our approach offers a unique strategy to capture and encode environmental source data into spikes and enables efficient sensing of multiple information in parallel processing for the application of adaptive sensory systems.

## 4 Experimental Section

### Fabrication of Two‐Terminal VO_2_ Threshold Switch

Epitaxial VO_2_ films with a thickness of 10 nm were grown on a (001)‐oriented TiO_2_ single‐crystal substrate at 300 °C under O_2_ pressure of 16 mTorr by pulsed laser deposition (PLD) in a chamber with base pressure of 10^−6^ Torr. Rotating targets synthesized by a conventional solid‐state reaction were irradiated by a KrF excimer laser (λ = 248 nm, Coherent Compex Pro 102F) at a fluence of ≈1 J cm^−2^ and a repetition rate of 1 Hz. After growth, the sample was cooled to room temperature at 20  °C min^−1^. To form a 35 µm × 100 µm channel area of VO_2_ thin films, standard photolithography, and single‐step wet etching were carried out using photoresist AZ 5214E (Merck), MA6 mask aligner (SUSS MicroTec.) and H_2_O_2_ solution (Merck). Subsequently, 100 nm‐thick Pt electrodes were patterned on top of VO_2_ thin films via radio frequency (RF) magnetron sputtering and a lift‐off process.

### Thin Film Characterization

Temperature‐dependent sheet resistance was obtained using the Van der Pauw method to characterize the metal‐insulator transition characteristics of the VO_2_ epitaxial films. The high crystal quality of VO_2_ epitaxial films was confirmed by symmetrical θ‐2θ X‐ray diffraction scans using a Bruker D8 Discover HRXRD with Cu Kα1 radiation (λ = 0.15406 nm). The absorption spectra of the VO_2_ epitaxial film were measured using a Perkin Elmer Lambda 750S UV–vis–NIR spectrophotometer. The thickness of the VO_2_ channel and Pt electrode of the switching devices were measured using X‐ray reflectometry (XRR). The plane view image of the two‐terminal threshold switch was collected by XL30 field emission gun scanning electron microscope (XL30. FEG SEM).

### Electrical Measurements

The VO_2_ threshold switch was connected to an external circuit to complete Pearson–Anson circuits for electrical oscillation output. Then, the device was placed in a temperature‐variable chamber probe station equipped with a semiconductor device analyzer (B1500A, Agilent) and a digital oscilloscope (DSOX3024T, Keysight). A source measurement unit (SMU) equipped in B1500A was used to source and read electrical signals in the entire circuit for current–voltage and current–voltage–time measurements, and DSOX3024T was used for high‐speed oscillation output (Figure S11, Supporting Information). To investigate the photothermal effect on an artificial nociceptor based on the Mott oscillator, the device was uniformly exposed using a broadband infrared mirror module (λ  = 750–1050 nm) along with UV (λ = 250–385 nm) and visible (λ = 385–740 nm) mirror modules, using a mercury xenon light source (Asahi Spectra, Max‐350, 300 W) at room temperature. MAX‐350 was set to emit controlled light emission via MAX‐350‐S‐N software (Asahi Spectra).

## Conflict of Interest

The authors declare no conflict of interest.

## Author Contributions

J.S. and P.H. conceived the idea and designed the study; P.H. performed the film deposition, X‐ray diffraction, device processing, and optoelectronic measurements with the assistance from D.Y., M.Y., and Y.P., and the guidance from J.S., J.S., and P.H. wrote the manuscript and all the authors commented on it. J.S. directed the overall research

## Supporting information



Supporting Information

## Data Availability

The data that support the findings of this study are available from the corresponding author upon reasonable request.
