# Supplementary material for: Artificial Photothermal Nociceptor Using Mott Oscillators
Source: Adv Sci (Weinh). 2024 Dec 18;12(6):2409353. doi: 10.1002/advs.202409353 (PMC11809409; doi:10.1002/advs.202409353)
Supplement: Supplementary file 1 — Supporting Information [file ADVS-12-2409353-s001.pdf]

ADVANCED SCIENCE
Open Access

Supporting Information

for *Adv. Sci.*, DOI 10.1002/advs.202409353

Artificial Photothermal Nociceptor Using Mott Oscillators

*Pyeongkang Hur, Daseob Yoon, Minwook Yoon, Yunkyu Park and Junwoo Son**

Supporting information for

Artificial photothermal nociceptor using Mott oscillators

Pyeongkang Hur[1)], Daseob Yoon[2)], Minwook Yoon[3), 4)], Yunkyu Park[1), #], Junwoo Son[3), 4), 5) *]

1) Department of Materials Science and Engineering, Pohang University of Science and Technology (POSTECH), Pohang 37683, Republic of Korea
2) Department of Electrical Engineering, Pukyong National University, Busan 48513, Republic of Korea
3) Department of Materials Science and Engineering, Seoul National University, Seoul 08826, Republic of Korea
4) Research Institute of Advanced Materials, Seoul National University, Seoul 08826, Republic of Korea
5) Institute of Applied Physics, Seoul National University, Seoul 08826, Republic of Korea

[#] Present address: Materials Science and Technology Division, Oak Ridge National University, TN, 37830, United States

* junuson@snu.ac.kr

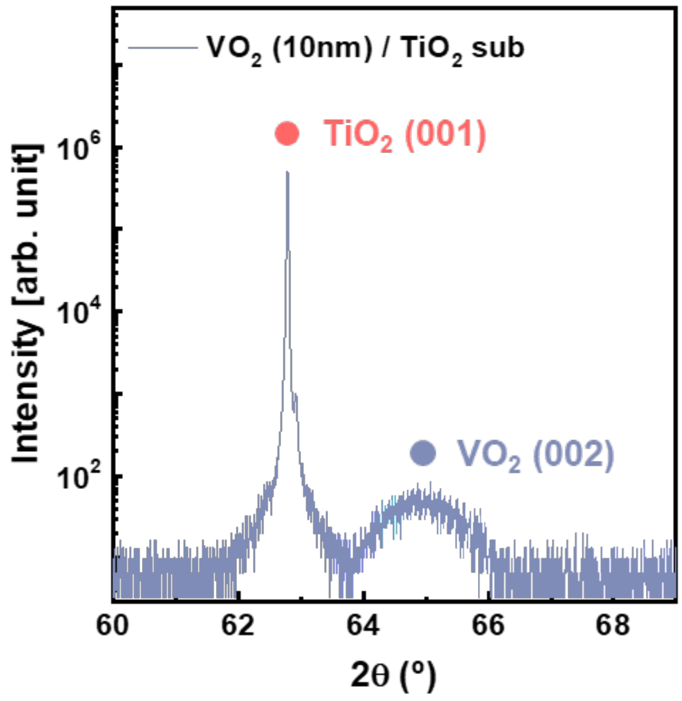

Figure S1 | 2theta-omega scan of 10-nm-thick VO_2 epitaxial films grown on a (001)-oriented TiO_2 single-crystal substrate.

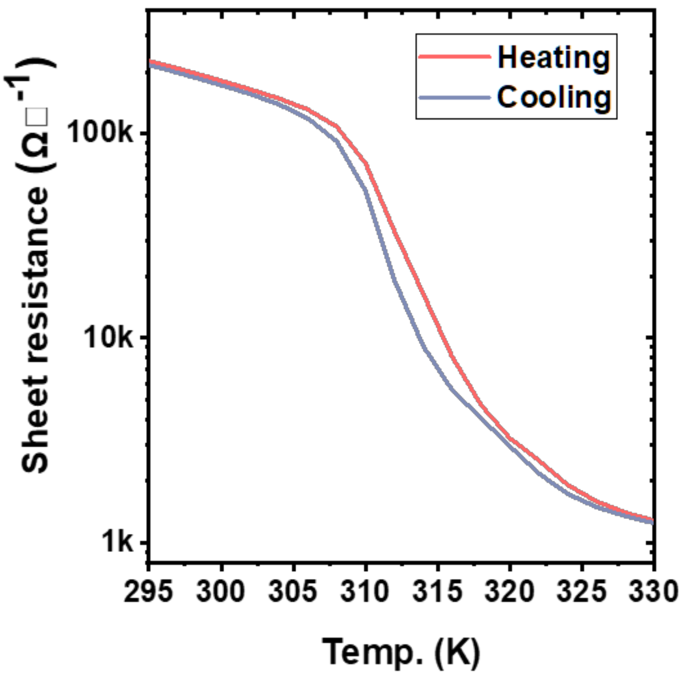

Figure S2 | Temperature dependent sheet resistance of VO_2 epitaxial films. The VO_2 epitaxial films exhibit a temperature-dependent metal-insulator transition at 310 K, enabling the demonstration of threshold switching at 298 K

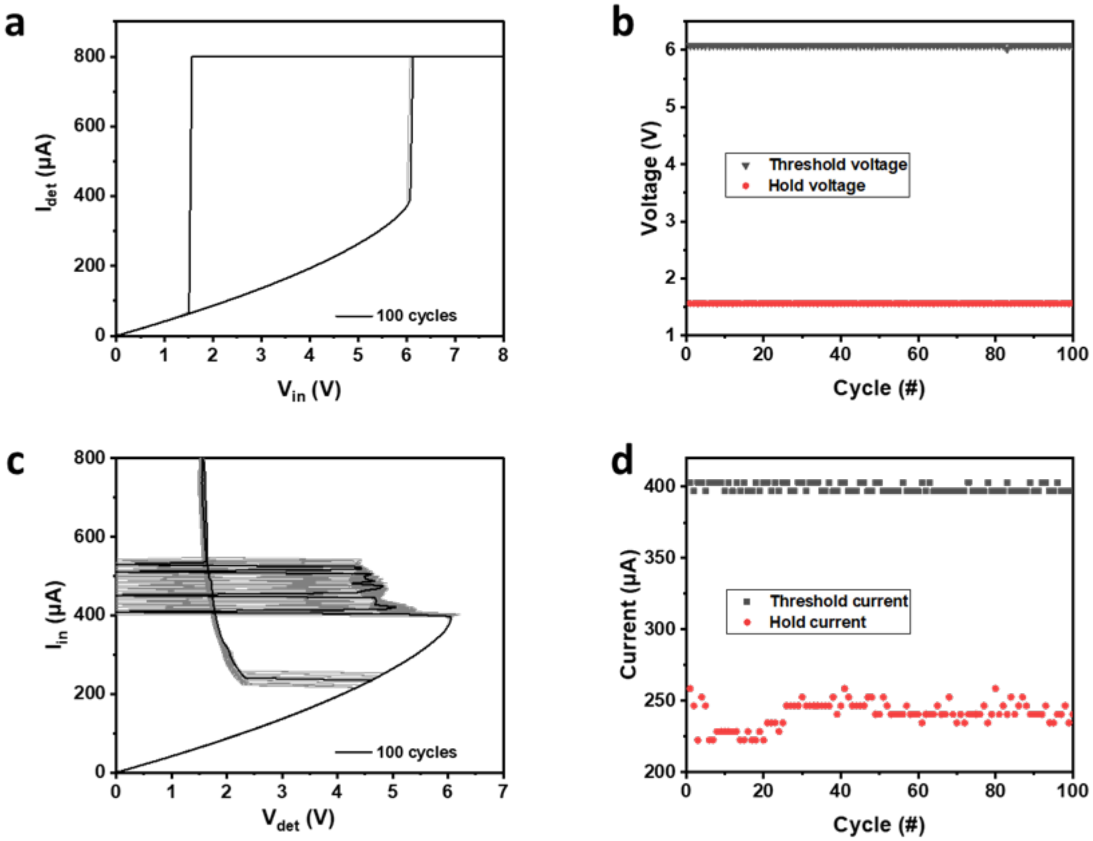

Figure S3 | Cycle-to-cycle variation of the VO_2 devices a, I-V curves recorded in V-mode over 100 switching cycles; **b,** V_{th} and V_{hold} values measured in V-mode across 100 cycles; **c,** I-V curves recorded in I-mode over 100 cycles; **d,** I_{th} and I_{hold} values measured in I-mode across 100 cycles.

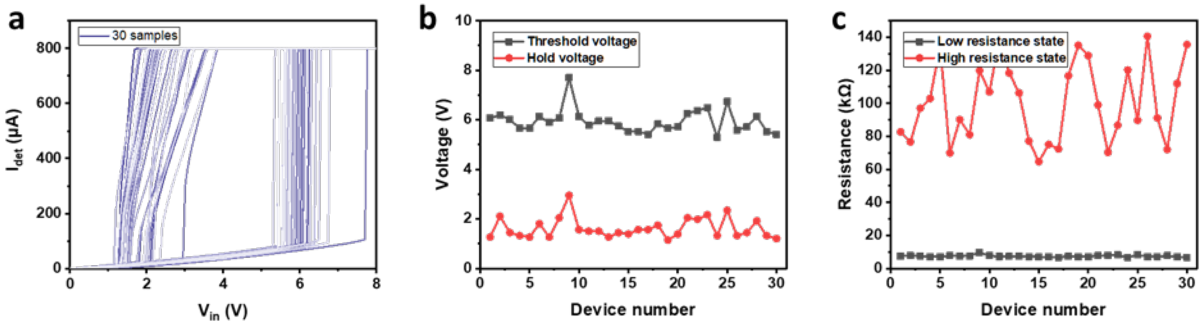

Figure S4 | Device-to-device variation of the VO_2 devices. a, I–V curves measured in voltage mode for 30 samples; **b**. V_{th} and V_{hold} values measured in voltage mode for 30 samples; **c**. Resistance values in the low resistance state and high resistance state obtained from (**a**)

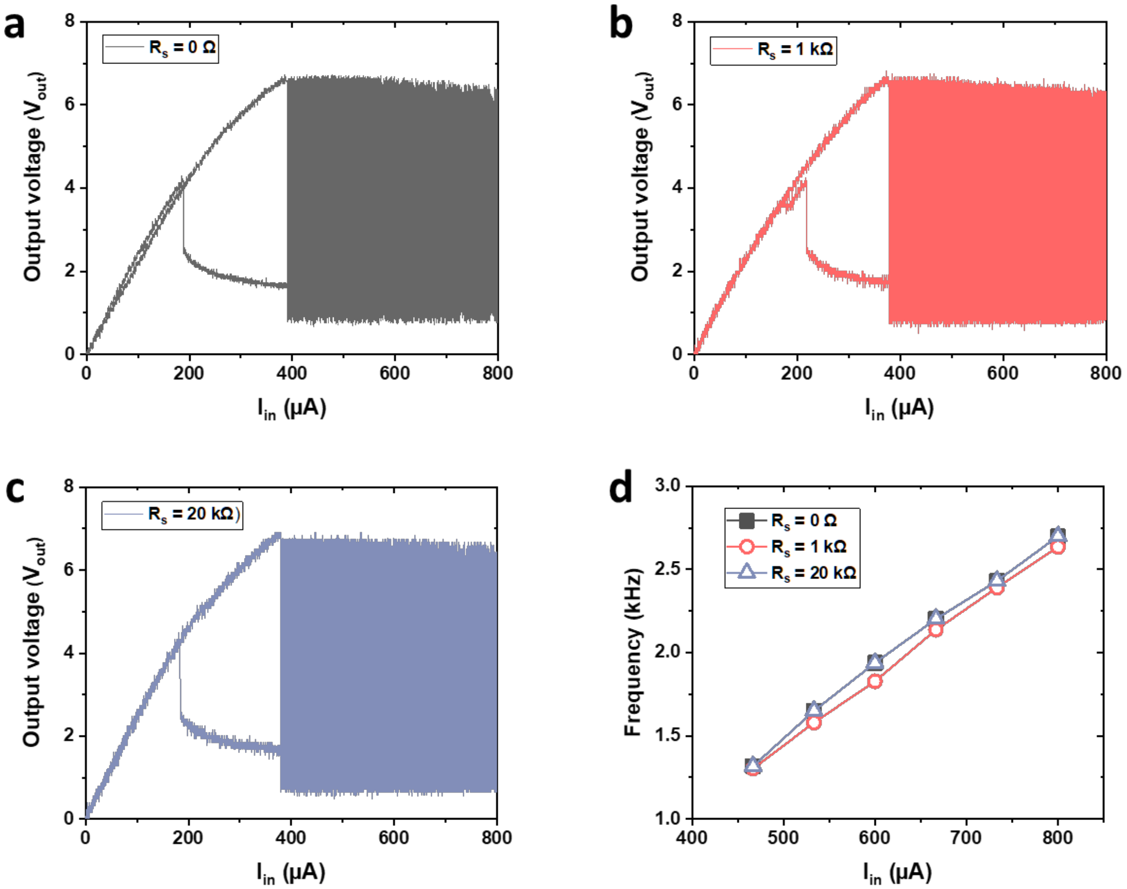

Figure S5. The negligible effect of series resistor connection on the output frequency. a–c, I-mode I–V output characteristics of VO_2 oscillators: **(a)** no resistor, **(b)** 1 kΩ series resisto**r, (c)** 20 kΩ series resistor. **(d)** Current-dependent frequency output for each sample.

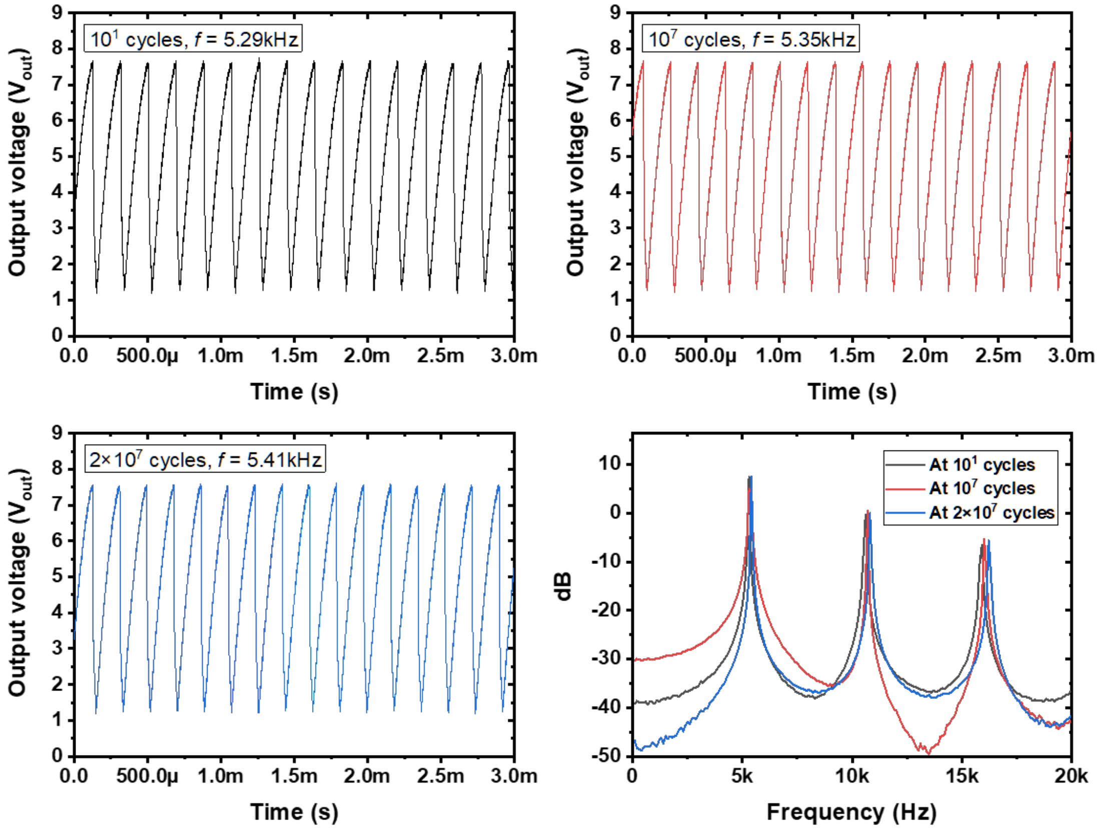

Figure S6 | Endurance of the VO_2 oscillator. Consistent spike output observed over 10^7 cycles, demonstrating the oscillator's long-term stability. The power spectrum, derived from output spikes within a 10 ms interval, shows a stable signal with distinct peaks at specific frequencies, indicating reliable operational consistency.

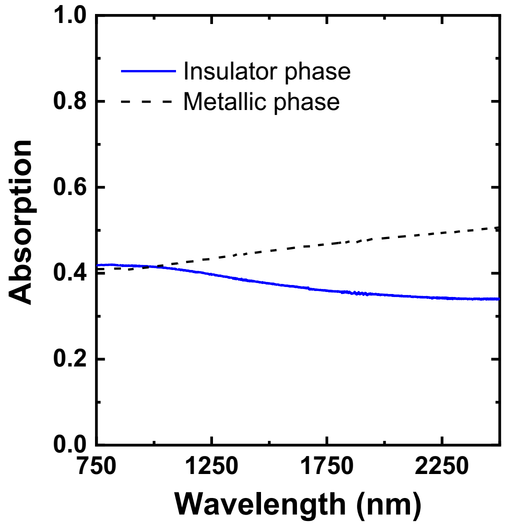

Figure S7 | The absorption spectra of VO_2 thin film across the 750 ~ 2500 nm range

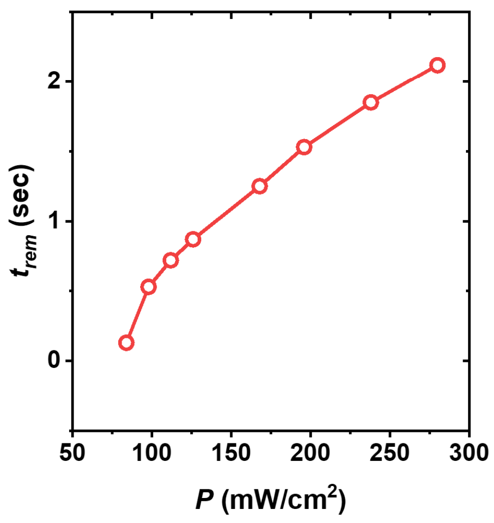

Figure S8 | The duration of remnant spike (t_{rem}) increasing with power (P). Input residual heat needs to be released from VO_2 layers even after radiation input ceases, which causes persistent spike generation due to gradual thermal release.

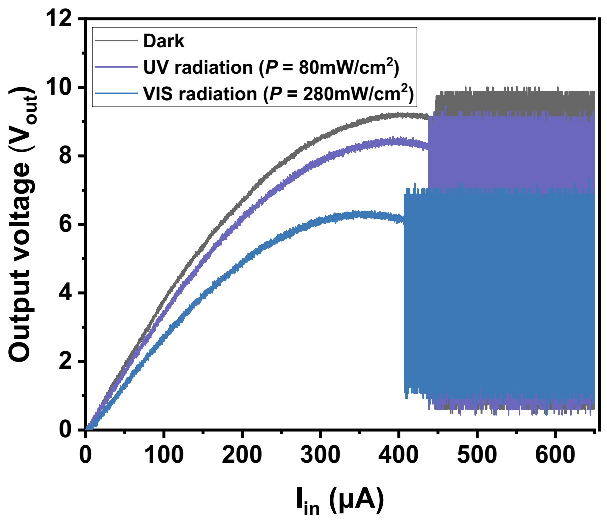

Figure S9 | Current-triggered threshold switching in VO_2 under exposure to ultraviolet and visible light radiation. Exposure to ultraviolet (P = 80 mW/cm^2) and visible light (P = 280 mW/cm^2) reduced the threshold current to 435 μA and 414 μA, respectively, and decreased the threshold $V_{out,\ th}$ to 8.16 V and 6.32 V.

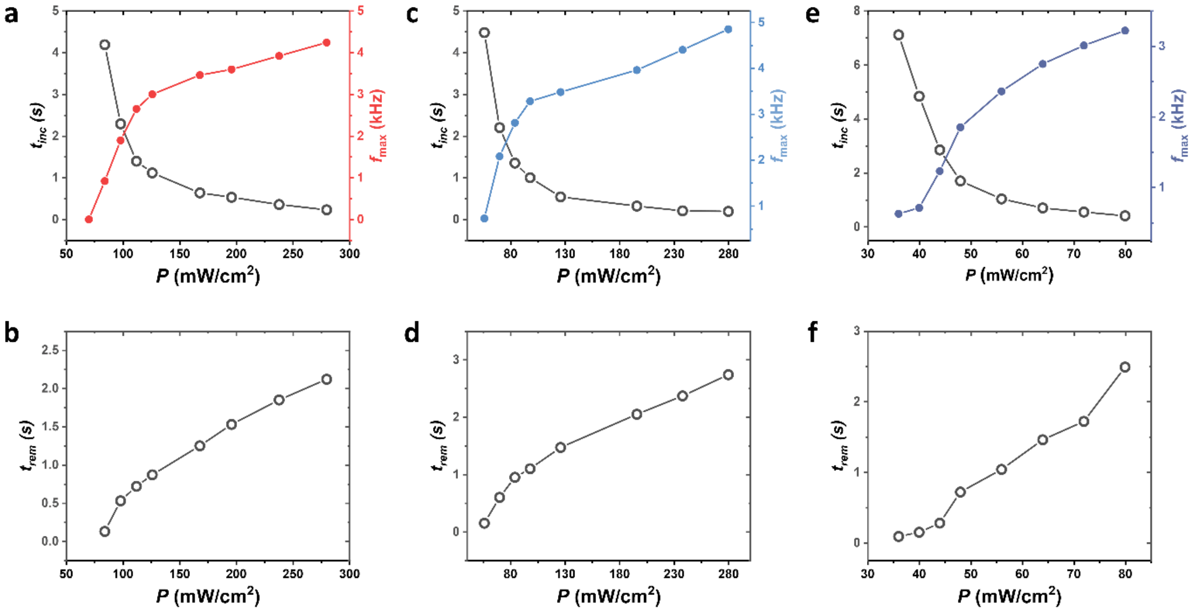

Figure S10. Variation in the parameters of the spike train in response to 10-second optical pulses of different wavelengths and *P* (power intensity). The dependence of t_{inc} and f_{max} on P is shown in **a, c** and **e**, while the dependence of t_{rem} on P is shown in **b, d** and **f** for infrared, visible, and ultraviolet photo-stimulation, respectively.

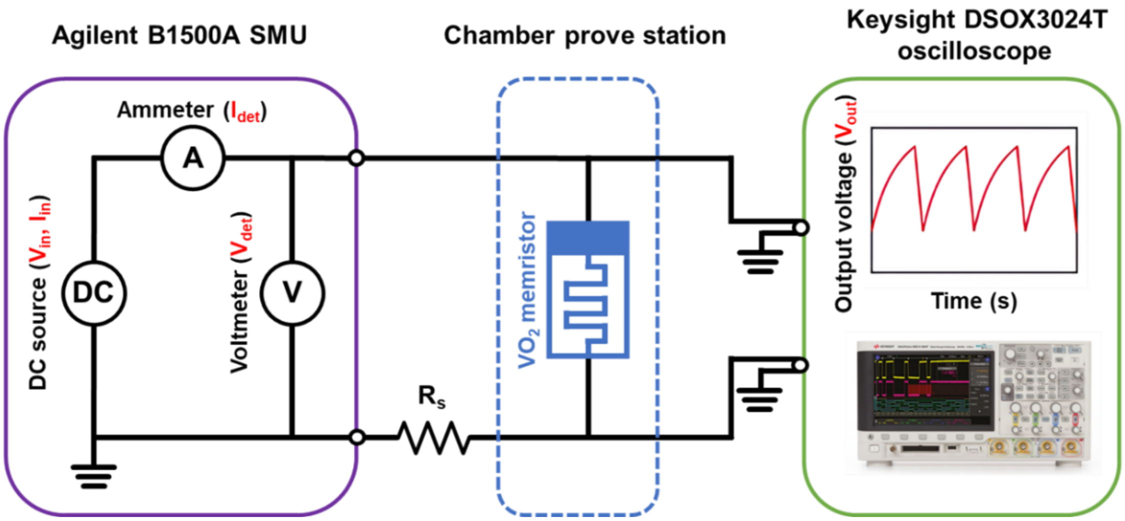

Figure S11 | Experimental setup for measuring the behavior of the Mott-oscillator. The VO_2 device is positioned in a chamber probe station to facilitate connection to an external circuit, which includes an Agilent B1500 source measure unit and a KEYSIGHT DSOX3024A oscilloscope.
